# Previous Homologous and Heterologous Stress Exposure Induces Tolerance Development to Pulsed Light in *Listeria monocytogenes*

**DOI:** 10.3389/fmicb.2016.00490

**Published:** 2016-04-08

**Authors:** Victoria Heinrich, Marija Zunabovic, Alice Petschnig, Horst Müller, Andrea Lassenberger, Erik Reimhult, Wolfgang Kneifel

**Affiliations:** ^1^Department of Food Science and Technology, Institute of Food Science, University of Natural Resources and Life SciencesVienna, Austria; ^2^OFI-Austrian Research Institute for Chemistry and TechnologyVienna, Austria; ^3^Department of Nanobiotechnology, Institute of Biologically Inspired Materials, University of Natural Resources and Life SciencesVienna, Austria

**Keywords:** kinetic analysis, *Listeria monocytogenes*, pulsed light, tolerance development, TEM, preservation technology

## Abstract

As one of the emerging non-thermal technologies, pulsed light (PL) facilitates rapid, mild and residue-free microbial surface decontamination of food and food contact materials. While notable progress has been made in the characterization of the inactivation potential of PL, experimental data available on the tolerance development to the same (homologous) stress or to different (heterologous) stresses commonly applied in food manufacturing (e.g., acid, heat, salt) is rather controversial. The findings of the present study clearly indicate that both the homologous tolerance development against PL as well as the heterologous tolerance development from heat to PL can be triggered in *Listeria monocytogenes.* Further, conducted kinetic analysis confirmed that the conventionally applied log-linear model is not well suited to describe the inactivation of *L. monocytogenes*, when exposed to PL. Instead, the Weibull model as well as the log-linear + tail model were identified as suitable models. Transmission electron microscopic (TEM) approaches allow suggestions on the morphological alterations in *L. monocytogenes* cells after being subjected to PL.

## Introduction

Research and development have increasingly focused on the substitution of conventionally used heat treatments like sterilization and pasteurization with novel and mild decontamination techniques in order to meet consumer expectations and microbiological safety criteria of foods (Aymerich et al., 2008; Sofos, 2008; Havelaar et al., 2010; Rajkovic et al., 2010; Weiss et al., 2010; Knorr et al., 2011). Hence, technologies like mild heat treatments, high pressure processing, pulsed electric field, weak organic acids and aqueous chlorine dioxide treatments, pulsed light (PL) as well as preservation by combined processes (hurdle technology) have been subjected to thorough investigation of the underlying mechanisms of microbial inactivation. Further, effectiveness, influence on the chemical, physical, and mechanical product characteristics and fulfillment of the requirements for industrial application (e.g., economic, ecologic factors and compliance to the respective legal background) were analyzed in the past (Leistner and Gorris, 1995; Butz and Tauscher, 2002; Sofos, 2005; Rajkovic et al., 2010; Ortega-Rivas, 2012).

Especially, PL, a non-thermal technology approved by the U. S. Food and Drug Administration (FDA) making progress to broad industrial application, is of highest interest for food business operators since it allows for a fast, mild and residue-free decontamination. In its basics, the PL technology comprises the generation of short-duration, high-power pulses of broad-spectrum (180–1100 nm) light via a Xenon flash lamp. The main germicidal action is, however, mainly restricted to the UV region of the light. When applying PL, it must be considered that the efficiency of the treatment is strongly influenced by the three main factors, namely the nature of the treated matrix (transparency or opacity, surface characteristics, and composition), microbial contamination (microorganism, physiological state, population density, and growth characteristics) and the process parameters (spectrum, geometry, and set up) chosen (Dunn et al., 1989; Food and Drug Administration [FDA], 1996; Palmieri and Cacace, 2005; Wang et al., 2005; Heinrich et al., 2016).

So far, notable progress has been made in describing the inactivation of diverse microorganisms, including pathogens of current concern like *Listeria monocytogenes*, *Escherichia coli* O157:H7, *Salmonella* spp., and *Campylobacter* spp., in or on different food and food contact matrices and under various PL process conditions (Heinrich et al., 2016). However, research concerning the possibility of the surviving microbial fractions to develop tolerance to the same (homologous) stress or cross-tolerance to or from different (heterologous) stresses commonly applied in food manufacturing (e.g., acid, heat, NaCl) is still in its early phase. Research in this area may therefore directly contribute to current issues in risk assessment and particularly in the analysis and management of hazards which arise with the substitution of conventionally used heat treatments in the food industry (Archer, 1996; Lou and Yousef, 1997; Hill et al., 2002; Gómez-López et al., 2005; Rajkovic et al., 2009, 2011).

Against this background, the present study aimed at contributing to a general agreement about the topic of tolerance development of microorganisms and, in particular, of *L. monocytogenes* strains against homologous, sub-lethal PL stress over an extended period of time as well as the stability of the formed tolerance in time. A further aim of the study was to perform kinetic analysis of the PL inactivation and investigate the possibility of forming cross-tolerance from heat stress to PL in *L. monocytogenes*. Transmission electron microscopy (TEM) was also utilized for the purpose of observing potential morphological alterations in *L. monocytogenes* cells as results of PL stress.

## Materials and Methods

### Bacterial Strains, Growth Conditions, and Inoculum Preparation

Information about the strains of *L. monocytogenes* used in the present study is summarized in **Table 1**. Bacterial stock cultures were kept at -80°C in Brain heart infusion (BHI) broth (Oxoid Limited, UK) with 20% glycerol added as cryogenic agent. Fresh cultures in their early stationary growth phase were prepared for each experiment by inoculating a loopful of the frozen culture in BHI and incubating at 37°C for 18 h. BHI was chosen as medium with optimal growth rates under controllable conditions based on the EURL technical guidance document (European Union Reference Laboratory for Listeria monocytogenes [EURL Lm], 2014). The resulting cell suspension of *L. monocytogenes* contained about 10^9^ colony forming units (CFU) mL^-1^. Microbial counts were performed by spreading 0.1 mL of the appropriate serial dilution in peptone water (PW; Merck, DE) on the non-selective medium Tryptone Soya Agar supplemented with 0.6% Yeast Extract (TSA + YE; Oxoid Limited, UK), incubating at 37°C and colony counting after 24 h.

**Table 1:** *Listeria monocytogenes* test strains used in this study.

Internal number	Strain designation	Origin and source
Li-10	ATCC 19115	Serotype 4b human isolate
Li-12	ATCC 19111	Serotype 1/2a; poultry
Li-16	ATCC BAA-751	Serotype 1/2b
Li-21	SLR 2249 Cornell university	*act*A deleted
Li-26	AFSSA 424 Li	Serotype 1/2a
Li-P492	L1/09	Outbreak strain
Li-P503	–	Cured pork neck after tumbling
Li-P535	–	Meat slicer environment
Li-P517	–	Drain meat processing after pasteurization
Li-P69	–	Fish factory environment

### Sample Preparation

Petri dishes having a diameter of 9 cm (Greiner Bio One, DE) were aseptically filled with 20 ± 1 g of the sterilized and cooled (55°C) TSA + YE medium, which resulted in a thickness of the agar layer of 11.7 ± 0.9 mm. This allowed for a constant distance between the surface of the inoculated petri dish and the PL emitting source. Surface inoculation was then done by serially diluting the cell suspensions in the respective sterile culture medium and spreading of a 0.1 mL aliquot of the selected dilutions on the TSA + YE medium. Inoculated samples were then left to dry at room temperature for 1 h at 20°C before PL treatment. Approximate microbial cell density of the samples was 10^6^ CFU cm^-2^. This cell density was chosen to avoid a possible shading effect, which is likely to occur from a microbial cell density equal or higher than 3^∗^10^7^ CFU cm^-2^ due to overlapping of cells (Gómez-López et al., 2005).

### Pulsed Light Treatment

For the PL treatment, the bench top device SteriPulse-XL RS-3000C (Xenon Corporation, MA) was used. This system has also been applied in previous studies (Keklik et al., 2009, 2010; Krishnamurthy et al., 2010; Haughton et al., 2011; Ringus and Moraru, 2013) and consisted of a sterilization time controller and a sterilization chamber, which was separated from the air-cooled lamp housing by a quartz window. The lamp housing held a single, cylindrical Xenon quartz lamp. Starting from an input voltage of 240, the system was capable of generating a radiant energy of 1.27 J cm^-2^ per pulse at 1.9 cm below the quartz window. Further, the pulse width was 360 μs, and after a default initial pulse, the pulse rate was 3 Hz.

Variation of the treatment intensity was facilitated by alternating the treatment time (s) and the distance between the quartz window and sample shelf. Of the 11 available shelf settings, settings 3 and 7 as counted from the quartz window were chosen for the tests. This resulted in distances of 4.5 and 9.6 cm from the quartz window. Samples centered on the shelf were treated for 0, 2, 5, 7, 10, 15 and 20 s. In order to allow intercomparison between different PL systems and to warrant result reproducibility, the total radiant energy received from the light source by the sample per unit area during the treatment time (fluence) was determined.

For the fluence (J cm^-2^) measurement the 3A-QUAD laser measurement sensor (Model No P/N 7z07934, Ophir Optronics Solutions Ltd) was used. The device was centered on the stainless steel shelf and recorded the energy levels over the respective times. The fluences associated with the respective treatment intensities are listed in **Table 2**.

**Table 2:** Fluence (J cm^-2^) measured at different PL treatment times and distances.

	Distance	
Time (s)	4.5 cm (shelf setting 3)	9.6 cm (shelf setting 7)
0	0 ± 0	0 ± 0
2	0.50 ± 0.17	0.46 ± 0.16
5	3.89 ± 0.25	2.58 ± 0.19
7	6.28 ± 0.23	3.79 ± 0.16
10	9.75 ± 0.20	5.59 ± 0.14
15	15.35 ± 0.17	8.32 ± 0.11
20	20.78 ± 0.15	10.84 ± 0.10

Surface temperature of the samples was measured before and right after the PL treatments using an infrared thermometer (Model 805, Testo, AT).

After PL treatment, 0.1 mL sterile physiological saline solution (0.85% NaCl) were brought onto the agar surface and the fluid was spread thoroughly. This was done to further minimize possible overestimation of inactivation levels due to the shading effect, where closely situated bacteria may develop as single colony (Gómez-López et al., 2005). Subsequently, the plates were brought to the dark of the 37°C incubator to minimize the chance of bacterial photoreactivation, a process where the endogenous enzyme photolyase in combination with visible light is capable of repairing PL caused DNA damage (Cleaver, 2003; Gómez-López et al., 2005). Colonies were then counted after 24 and 48 h and expressed as CFU cm^-2^. Since no significant increase in CFU was observed for the 48 h incubation, results are expressed only for the 24 h incubation samples.

Plates containing between 10 and 300 colonies were evaluated and the results expressed as CFU cm^-2^. The surviving fraction of *L. monocytogenes* was then expressed as log (N/N_0_), where *N* represents the microbial cell density (CFU cm^-2^) and *N*_0_ the initial microbial cell density (CFU cm^-2^).

To determine the sensitivity of *L. monocytogenes* strains (**Table 1**) to PL, the bacteria were treated at the lowest PL intensity of 0.46 J cm^-2^, an intensity where survival of *L. monocytogenes* was likely. The lower the degree of inactivation the more resistant the bacteria were. From the results, two *L. monocytogenes* strains were chosen for the tolerance tests.

### Tolerance Development to Homologous PL Stress

To investigate the ability of *L. monocytogenes* Li-16 and Li-P492 to develop tolerance against sub-lethal PL treatment, the stress protocol described by Gómez-López et al. (2005) was applied with slight modifications. Starting from three subcultures of each of the two tested *L. monocytogenes* strains in BHI, serial dilutions in BHI were spread onto TSA + YE. Subsequent to the sub-lethal PL treatment of 0.46 J cm^-2^, plates were incubated at 37°C for 48 h. After enumeration of the surviving fraction, one randomly selected colony from each subculture was transferred to BHI and cultured at 37°C for 24 h, plated and flashed again at the same conditions. This sequence of actions was repeated 20 times during a 46 days period. To compare control and stressed *L. monocytogenes* strains, resulting cultures were treated with fluences ranging from 0.46 to 20.78 J cm^-2^ (**Table 2**).

### Influence of Deep-Freeze Storage of *L. monocytogenes* Stains on PL Tolerance

To check the retention of the increased tolerance over time, control and tolerant strains were stored under deep-frozen conditions (-30°C) for 133 days and again subjected to fluences ranging from 0.46 to 20.78 J cm^-2^ (**Table 2**).

### Heat Stress and Cross-Tolerance Development to PL

In order to identify the potential of heat stressed cells of Li-P492 for the development of cross-tolerance to PL, the procedure of Lin and Chou (2004) was applied with slight modifications. Starting from a culture in the stationary growth phase, a 10 mL aliquot was mixed with 90 mL physiological saline solution (0.85% NaCl) in a 200 mL glass flask. Subsequently, the tube was submerged in a 47°C tempered, circulating water bath (Medingen W22, Preiss-Daimler, DE). The actual temperature of the cell suspension was measured using a thermometer (LLG- General-purpose thermometer, measuring range -10 to +100°C) in a reference flask containing physiological saline solution (0.85% NaCl) solely. After the short temperature adaption period of 5 min, the time for the 1 and 1.5 h heat stress was recorded as soon as the 47°C mark was reached. After the heat stress, the cell suspension was immediately cooled down in ice water for 1 min and subjected to PL treatment of 0.46 J cm^-2^.

### Statistical Analysis

The data obtained from the randomized trials (triplicate tests with threefold determination) were compared by Analysis of Variances (ANOVA) using IBM SPSS statistic software, version 21. The *post hoc* test chosen was the Bonferroni test. Statistical evaluations were based on a 95% confidence interval (α = 0.05).

### Inactivation Kinetics

Survival curves were obtained by plotting the logarithm of microbial cell density (CFU cm^-2^) against the treatment intensity, given in fluence (J cm^-2^). As the curves did not fit the classical log-linear approach, conventionally used for heat sterilization processes, the Geeraerd and Van Impe inactivation model-fitting tool (GInaFiT; Version 1.6), a freeware add-in for Microsoft Excel, was used to describe the inactivation curves of the pathogen (Geeraerd et al., 2005). The two additional mathematical models chosen were the log-linear + tail and the Weibull model, as they more closely fit the data.

The log-linear model assumes that all individuals in a population have equal sensitivity to the applied heat treatment. This means that a linear relationship between the death of an individual and the random chance of sufficiently affecting a key molecule or “target” via the treatment is given (Bigelow and Esty, 1920; Cole et al., 1993; Geeraerd et al., 2005). In static conditions, this can be calculated using Eq. (1).

(1) $$\log_{10}\left(N\right)=\log_{10}\left(N\left(0\right)\right)-\frac{t}{D}=\log_{10}\left(N\left(0\right)\right)-\frac{k_{\max}t}{In\left(10\right)}\,$$

In this context *N* describes the microbial cell density (CFU cm^-2^), *N*(0) stands for the initial microbial cell density (CFU cm^-2^), *k*_max_ represents the first order inactivation constant (1/time unit) and *D* describes the decimal reduction time (time unit; Bigelow and Esty, 1920; Geeraerd et al., 2005).

Authors repetitively highlight the inactivation curve of microorganisms subjected to PL to be non-log-linear (Rowan et al., 2015). More particularly, three phases, namely shoulder, log-linear phase and tailing region can be recognized due to the existence of resistant sub-populations (Cerf, 1977). Since log-linear behavior with and without shoulder and/or tailing can be observed, the GInaFiT add-in covers a log-linear model with shoulder and/or tailing (Geeraerd et al., 2005). Setting the shoulder or the residual population density in Eq. (2) equal to zero leads, in static conditions, to the reduced models of log-linear + shoulder and log-linear + tail, respectively (Geeraerd et al., 2000, 2005).

(2) $$\log_{10}\left(N\right)=\log_{10}\left[\left(10^{\log_{10}\left(N\left(0\right)\right)}-10^{\log_{10}\left(N_{res}\right)}\times e^{{-k}_{\max}t}\times \left(\frac{e^{k_{\max}S_{1}}}{1+(e^{k_{\max}S_{1}})\times e^{-k_{\max}t}}\right)+10^{\log_{10}\left(N_{res}\right)}\right)\right]\,$$

Herein, *N* describes the microbial cell density (CFU cm^-2^), *N*(0) represents the initial microbial cell density (CFU cm^-2^), *N*_res_ stands for the residual population density (CFU cm^-2^), *k*_max_ describes the specific inactivation rate (1/time unit), and *S*_1_ is a parameter representing the shoulder (time unit). In the case of the log-linear + tail model, setting the shoulder equal to zero results in Eq. (3), (Geeraerd et al., 2000, 2005).

(3) $$\log_{10}\left(N\right)=\log_{10}\left[\left(10^{\log_{10}\left(N\left(0\right)\right)}-10^{\log_{10}\left(N_{res}\right)}\times e^{{-k}_{\max}t}+10^{\log_{10}\left(N_{res}\right)}\right)\right]\,$$

The Weibull model assumes that the entire microbial population is not equally resistant to the lethal treatment and as a consequence, that each individual is not destroyed at the same time during the treatment. As a result, the Weibull survival curve is the cumulative form of a temporal distribution of lethal events where each individual is inactivated at a specified time (Peleg and Cole, 1998; Mafart et al., 2002; Geeraerd et al., 2005). This spectrum of tolerances is depicted in Eq. (4), (Peleg and Cole, 1998; Mafart et al., 2002).

(4) $$\log_{10}(N)=\log_{10}\left(N\left(0\right)\right)-{\left(\frac{t}{\delta }\right)}^{P}\,$$

In this context *N* is the microbial cell density (CFU cm^-2^), *N*(0) stands for the initial microbial cell density (CFU cm^-2^), *t* describes the inactivation time (time unit), δ is a scale parameter and can be denoted as the time (time unit) that leads to the first decimal reduction of the surviving population if *p* = 1 and *p*(-) describes the shape parameter. A *p* > 1 describes a convex, while a *p* < 1 describes a concave shaped survival curve. A *p* = 1 corresponds to the log-linear shape (Mafart et al., 2002; Geeraerd et al., 2005).

Parallel to Huang (2009), the curves were fitted separately and the accuracy of the kinetic models was compared on basis of the automatically in the GinaFiT add-in generated, statistical parameter square root of the mean squared error (RMSE). The statistical measure is regarded as a simple and informative measure of goodness of fit for both, linear and non-linear models and gives the “average” discrepancy between the (transformed) observed data and their predicted pendants. So, the RMSE in regard of the precision of the observed data is useful to determine if a model fits the data (Ratkowsky, 2003). The RMSE can be calculated using Eq. (5).

(5) $$RMSE=\sqrt{\frac{1}{N}\overset{N}{\underset{i=1}{\Sigma }}}{\left(x_{i}-{\overset{-}{x}}_{i}\right)}^{2}\,$$

Herein, *N* is the number of data points, *x*_i_ stands for the logarithmic cell density (CFU cm^-2^) observed and *x*_i_ is the logarithmic cell density estimated by the model (Ratkowsky, 2003; Huang, 2009).

Subsequently, analysis of variance (ANOVA) was conducted to compare the mean RMSE among the different models. The Bonferroni *post hoc* test was used to group the means of RMSE based on a 95% confidence interval (α = 0.05). The statistical analysis was conducted using IBM^®^ SPSS^®^ statistic software, version 21.

### Transmission Electron Microscopy (TEM)

#### Sample Preparation

*Listeria monocytogenes* strain Li-P69 was grown overnight at 37°C for 18 h in BHI. An aliquot of 0.1 mL of the bacterial suspension (1:10) was plated onto Gelerite Plates. Gelerite was preferred instead of Agar-based media due to its translucent nature and the solid surface structure in order to obtain a high amount of the bacterial suspension for TEM analysis. In brief, Gelerite plates were prepared from 100 mL of BHI and 7 g Gelerite (Carl Roth, DE) and further autoclaved. Plates were poured and solidified at room temperature before inoculation. After inoculation, plates were stored at room temperature for 1 h prior PL treatment. Li-P69 was treated for 3 and 5 s at a distance of 9.6 cm, which resulted in fluences of 1.20 and 2.58 J cm^-2^. After the treatment, plates were flushed with physiological saline solution (0.85% NaCl) and transferred to a micro reaction tube followed by a centrifugation step at 7.000 rpm for 5 min. Control cells were processed by the same protocol.

#### Transmission Electron Microscopy (TEM)

Chemicals are, if not stated otherwise, from Sigma–Aldrich, Austria. All steps of the embedding process were performed under a fume hood on a shaker at 100 rpm.

The bacterial pellet was mixed with 50 μl of 2% Agarose in distilled water and cut in stripes after solidification. Fixation was performed using a fixation solution containing 2.5% Paraformaldehyde, 2.5% Glutaraldehyde, and 2.5 mM CaCl_2_ in 0.1 M Na-Cacodylat in distilled water pH 7.4 plus 1% Tannic Acid for 2 h at room temperature, followed by another fixation step without Tannic Acid over night at 4°C. Afterwards, a threefold washing step with 0.1 M Na-Cacodylat was carried out. Postfixation with 1% OsO_4_ in 1.5% Ferricyanid solution followed by an additional step in 1% OsO_4_. After fixation, the cells were rinsed three times with distilled water and dehydrated in a graded ethanol series from 70 to 100%. The cells were sequentially infiltrated with LR-White Resin (Fluka, Germany), transferred into gelatin capsules size “00” and polymerized at 60°C for 40 h. The blocks were hand trimmed with a razor blade and slices of 70 nm were cut using a Leica Ultracut UC7. Slices were placed on 150 mesh copper grids and examined using a Tecnai G20 Transmission Electron Microscope with an acceleration voltage of 160 kV. Two replicates were performed per treatment time.

## Results And Discussion

Novel and mild inactivation technologies open up new possibilities in meeting legal requirements in terms of food safety as well as modern consumer demands (Aymerich et al., 2008; Sofos, 2008; Havelaar et al., 2010; Rajkovic et al., 2010; Weiss et al., 2010; Knorr et al., 2011). However, incomplete inactivation and sub-lethal damage of the target microorganisms is a critical hazard that cannot be neglected, as it not only endangers microbial quality and safety of the products but entails the danger of modified properties of the surviving bacterial sub-populations. In this regard, (cross) tolerance and modified virulence characteristics is one of the major concerns in the scientific community and among food business operators (Lou and Yousef, 1997; Rowan, 1999; Rowan et al., 1999; Hill et al., 2002; Francois et al., 2006; Rajkovic et al., 2009, 2010). Thereby, bacteria response to inimical environmental factors such as temperature, pH, osmolarity or oxidative stress by exhibiting diverse physiological and molecular responses (Archer, 1996; Rowan, 1999; Rajkovic et al., 2009). Such effects have, for example, been shown for technologies like high pressure processing and pulsed electric field, and it seems that increased tolerance is not an abrupt process but a result of repetitive exposure to sub-lethal treatments. Additionally, tolerance development was found to be species-, strain- as well as treatment- specific (Yousef and Courtney, 2003; Rajkovic et al., 2010).

In recent years, some research has been conducted aiming at assessing whether and to what extent (cross) tolerance development occurs in (pathogenic) microorganisms exposed to PL. Nevertheless, the experimental data are rather controversial and there is no general agreement on this topic (Marquenie et al., 2003; Gómez-López et al., 2005; Rajkovic et al., 2009, 2011; Massier et al., 2011; Bradley et al., 2012; Uesugi et al., 2013).

### Tolerance Development to Homologous PL Stress and Influence of Deep-Freeze Storage

By subjecting the *L. monocytogenes* strains listed in **Table 1** to a PL stress of 0.46 J cm^-2^, a considerable reduction of initial microbial cell density (log CFU cm^-2^) was obtained. For *L. monocytogenes* the inactivation levels ranged from 3.15 ± 0.04 to 5.43 log CFU cm^-2^. Details on initial counts as well as the inactivation levels are listed in **Table 3**. *L. monocytogenes* Li-16 and Li-P492 strains were chosen as indicator organisms for the subsequent experiments. The main reason for this selection lies in the fact that the strains were among the most tolerant to PL and that *L. monocytogenes* is a pathogen of current concern in the food industry (The Rapid Alerst System for Food, and Feed [RASFF], 2014; European Food Safety Authority, and European Centre for Disease Prevention, and Control [EFSA and ECDC], 2015).

**Table 3:** Mean reduction (log CFU cm^-2^) and standard deviation of *L. monocytogenes* viable counts on Tryptone Soya Agar supplemented with Yeast Extract when exposed to sublethal PL stress of 0.46 J cm^-2^.

Internal number	Inactivation ± SD (log10 CFU cm^-2^)
Li-10	5.02 ± 0.92
Li-12	5.32 ± 0.17
Li-16	3.35 ± 0.12
Li-21	4.70 ± 0.72
Li-26	>5.24^∗^
Li-P492	3.15 ± 0.04
Li-P503	>5.43^∗^
Li-P535	4.98 ± 0.35
Li-P517	4.92 ± 0.24

*^∗^Variance could not be calculated when inactivation exceeded the detection limit.*

**Table 4** summarizes the mean logarithmic reduction of microbial cell density (CFU cm^-2^) of *L. monocytogenes* Li-16 and Li-P492 inoculated on TSA + YE medium when applying fluences ranging from 0.46 to 20.78 J cm^-2^ (**Table 2**). The PL treatment was performed on control strains, strains that exhibited homologous stress over an extended period of time and stressed strains stored under deep-freeze conditions over an extended period of time.

**Table 4:** Mean reduction (log CFU cm^-2^) of viable counts of *L. monocytogenes* Li-16 and Li-P492 on Tryptone Soya Agar supplemented with Yeast Extract when exposed to different PL treatment intensities.

	Li-16			Li-P492		
Fluence (J cm^-2^)	Control strain	Tolerant strain	Tolerant and stored strain	Control strain	Tolerant strain	Tolerant and stored strain
0 ± 0	0 ± 0	0 ± 0	0 ± 0	0 ± 0	0 ± 0	0 ± 0
0.46 ± 0.16	3.35 ± 0.12	1.98 ± 0.02	2.82 ± 0.03	3.15 ± 0.04	0.75 ± 0.01	1.84 ± 0.11
0.50 ± 0.17	3.34 ± 0.09	1.96 ± 0.12	2.85 ± 0.04	3.28 ± 0.03	0.82 ± 0.01	1.79 ± 0.25
2.58 ± 0.19	4.82 ± 0.01	2.67 ± 0.03	3.75 ± 0.25	3.80 ± 0.16	2.84 ± 0.03	2.99 ± 0.18
3.79 ± 0.16	5.01 ± 0.02	3.07 ± 0.16	4.81 ± 0.04	5.25 ± 0.02	3.03 ± 0.04	3.57 ± 0.10
3.89 ± 0.25	4.86 ± 0.02	2.75 ± 0.04	3.83 ± 0.23	4.07 ± 0.14	3.07 ± 0.11	3.10 ± 0.02
5.59 ± 0.14	5.32 ± 0.02	3.55 ± 0.35	5.34 ± 0.04	5.45 ± 0.04	3.86 ± 0.22	4.08 ± 0.16
6.28 ± 0.23	5.06 ± 0.01	3.41 ± 0.10	4.85 ± 0.01	5.22 ± 0.03	3.18 ± 0.04	3.46 ± 0.05
8.32 ± 0.11	5.50 ± 0.07	5.00 ± 0.02	5.56 ± 0.08	6.10 ± 0.03	5.16 ± 0.04	5.50 ± 0.09
9.75 ± 0.20	5.35 ± 0.03	3.67 ± 0.02	5.35 ± 0.04	5.49 ± 0.07	3.95 ± 0.20	4.11 ± 0.14
10.84 ± 0.10	5.92 ± 0.17	5.16 ± 0.05	6.08 ± 0.02	6.51^∗^	5.55 ± 0.06	6.00 ± 0.05
15.35 ± 0.17	5.70 ± 0.05	5.21 ± 0.04	5.70 ± 0.04	6.28 ± 0.03	5.25 ± 0.05	5.46 ± 0.09
20.78 ± 0.15	6.19 ± 0.05	5.76 ± 0.14	6.01 ± 0.13	6.51^∗^	5.78 ± 0.07	6.07 ± 0.19

**Mathematical model**	**RMSE**					

Weibull-type	0,446	0,804	0,534	1,068	0,454	0,653
Log-linear + tail	0,868	0,621^∗∗^	0,766	0,418^∗∗^	0,729	0,828
Log-linear	1,239	1,021	1,071	1,495	0,904	1,385

*Further, the goodness of fit parameter Root Mean Square Error (RMSE) of the log-linear, log-linear + tail as well as Weibull models, predicting the reduction of viable counts of L. monocytogenes, are given. ^∗^Maximum inactivation. ^∗∗^Cases where the Log-linear + tail model fitted the data better than the Weibull model.*

The statistical analysis revealed that the dependent variable reduction of microbial cell density was significantly (*p* = 0.01) influenced by the independent variables strain, condition and PL treatment intensity. Therefore, one result to emerge from the data is that, next to the treatment intensity, the strain specific nature of *L. monocytogenes* Li-16 and Li-P492 influences the sensitivity to PL. Further, strong evidence of tolerance development in both strains was found when applying homologous PL stress over an extended period of time. Interestingly, the tolerance retention seems to be time dependent. So, the level of tolerance declined considerably (*p* = 0.01) after the deep-freeze storage period.

**FIGURE 1:**
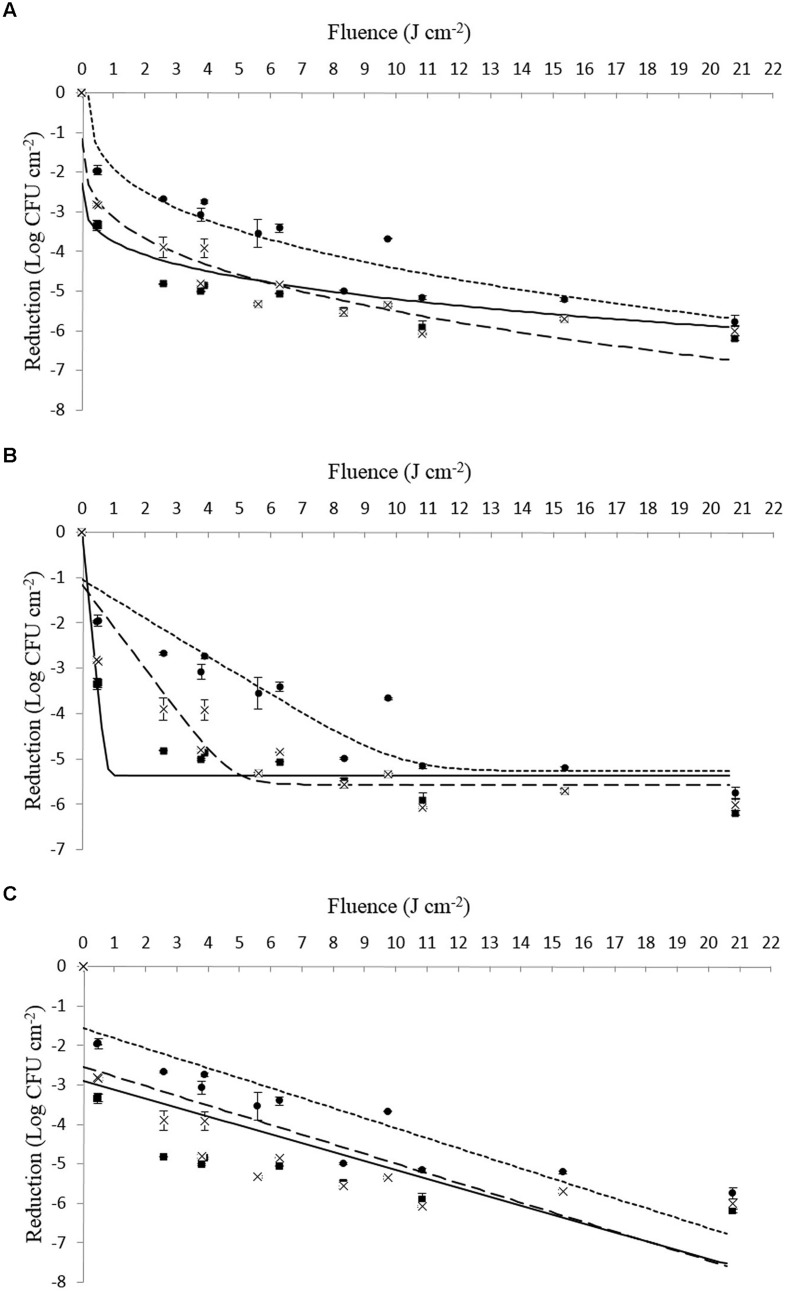
**(A–C)** Survival (log CFU cm^-2^) of viable counts of *Listeria monocytogenes* Li-16 inoculated on Tryptone Soya Agar supplemented with Yeast Extract when exposed to different PL treatment intensities (0.46 ± 0.16 to 20.78 ± 0.15 J cm^-2^). The symbols represent observed means for control (■), tolerant (●) as well as tolerant and stored (x) strains. The lines represent the respective mathematical inactivation models calculated on basis of the observed values for control (solid line), tolerant (dotted line) as well as tolerant and stored strains (dashed line). **(A)** Weibull-type model. **(B)** Log – linear + tail model. **(C)** Log – linear model.

**FIGURE 2:**
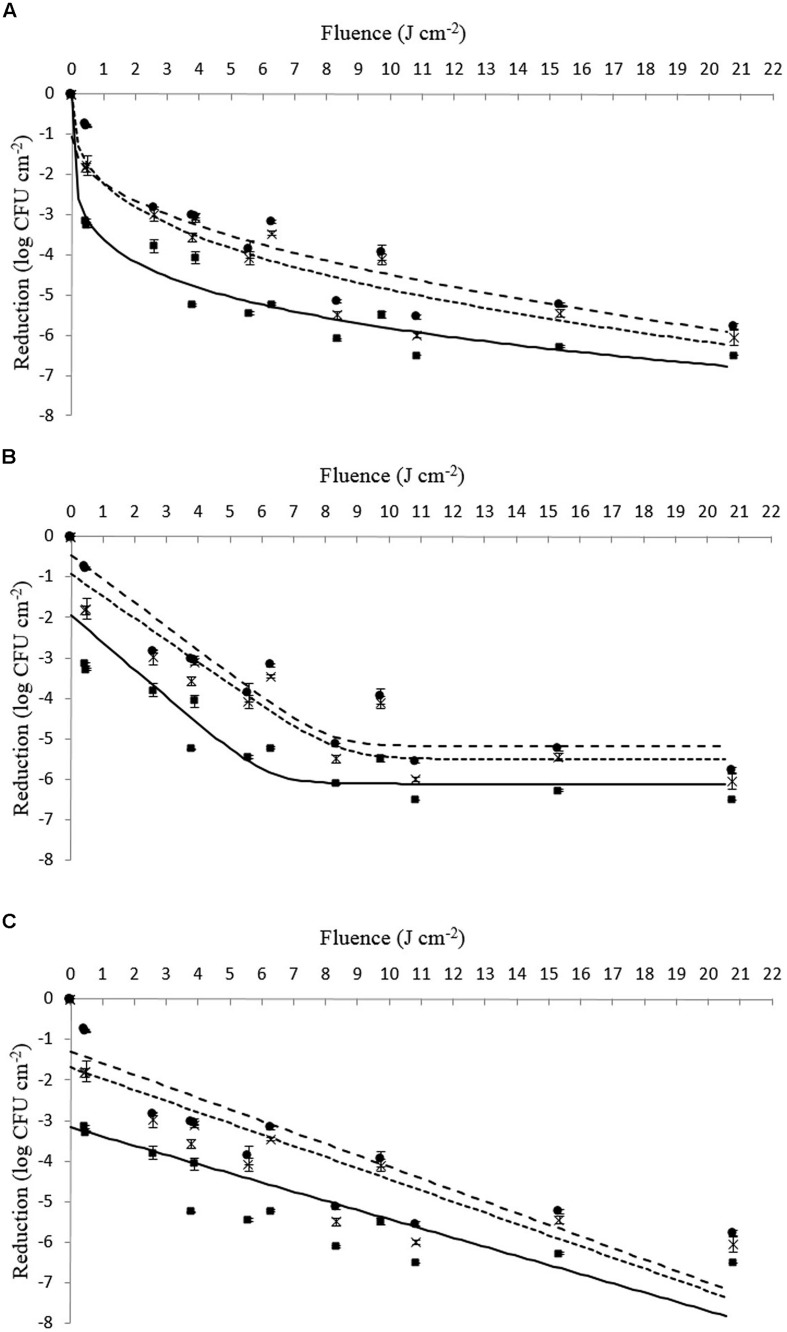
**(A–C)** Survival (log CFU cm^-2^) of viable counts of *L. monocytogenes* Li-P492 inoculated on Tryptone Soya Agar supplemented with Yeast Extract when exposed to different PL treatment intensities (0.46 ± 0.16 to 20.78 ± 0.15 J cm^-2^). The symbols represent observed means for control (■), tolerant (●) as well as tolerant and stored strains (x). The lines represent the respective mathematical inactivation models calculated on basis of the observed values for control (solid line), tolerant (dotted line) as well as tolerant and stored strains (dashed line). **(A)** Weibull-type model. **(B)** Log – linear + tail model. **(C)** Log – linear model.

On closer examination of the data presented in **Table 4** in combination with **Figures 1** and **2**, it can be seen that strains of Li-16 and Li-P492 exhibit a steep decrease in microbial cell density in the first half and progressive leveling-out of the curve in the second half of the PL treatment. The maximum reduction in microbial cell density was observed at the highest treatment intensity of 20.78 J cm^-2^ for control strains of Li-16 and Li-P492. Corresponding values are 6.19 ± 0.05 and 6.51 log CFU cm^-2^.

Referring to the statistical analysis of Li-16, the significant (*p* = 0.01) tolerance development due to homologous stress over an extended period of time resulted in a mean difference between the control and tolerant strains of Li-16 of 1.16 ± 0.02 log CFU cm^-2^. After deep-frozen storage, the mean difference (*p* = 0.01) between the control and the stored strains of Li-16 was still significant, but reduced to 0.32 ± 0.02 log CFU cm^-2^. The significant (*p* = 0.01), mean difference between stressed and stored strains of Li-16 whereas was 0.85 ± 0.02 log CFU cm^-2^.

Similarly, Li-P492 exhibited a significant (*p* = 0.01) mean tolerance development of 1.23 ± 0.02 and reduction after storage to 0.87 ± 0.02 log CFU cm^-2^. The mean difference between stressed and stored strains of Li-P492 whereas was reduced to 0.35 ± 0.02 log CFU cm^-2^, but also significant (*p* = 0.01).

Interestingly, the findings of the present study are not in line with previous research conducted by Marquenie et al. (2003), Gómez-López et al. (2005), and Uesugi et al. (2013). Gómez-López et al. (2005), for example, stated that after 13 cycles of PL treatment, re-cultivation and re-exposure of the surviving fractions to PL no tolerance of *L. monocytogenes* against PL was induced. Similarly, Uesugi et al. (2013) found that up to 10 cycles of PL treatment did not influence the tolerance behavior or growth kinetics of *L. monocytogenes*, *L. innocua*, and *E. coli*. Also, Marquenie et al. (2003) could not demonstrate tolerance development of conidia of *Botrytis cinerea* and *Monilinia fructigena* after 10 cycles of PL treatment.

However, the findings of the present study are to a large extent consistent with those of Rajkovic et al. (2009) and Massier et al. (2011), who also do not support the above authors’ conclusion of PL being a technology not selecting for resistant microorganisms. Rajkovic et al. (2009) for example detected a tolerance development of approximately 1 log CFU mL^-1^ in a four-strain mix culture of *L. monocytogenes* when applying homologous, sub-lethal PL stress over an extended period of time. At the end of this period, the authors were able to show that the strain with the lowest initial susceptibility to PL was predominant at the end of the experiment and concluded that the strain was able to overgrow more sensitive strains and further enhance its tolerance. Interestingly, a similar pattern of tolerance development (2 log CFU mL^-1^) and predominance of one strain at the end of the experiment was shown for *E. coli* O157:H7. Further, the increased tolerance against PL of *L. monocytogenes* and *E. coli* O157:H7 was shown to remain stable over 12 months storage at -75°C and tolerant strains exhibited considerable longer lag phase before the onset of the exponential growth when compared to the control strains. In a subsequent study, Rajkovic et al. (2011) were able to verify that – although the exact underlying mechanism of increased tolerance is not yet known – increased tolerance against PL significantly modifies the growth characteristics of the tested strains. This has, for example, also been shown for barotolerant *E. coli* (Hauben et al., 1997). Further, Rajkovic et al. (2011) did not report cross-tolerance development of PL tolerant strains toward heat and low pH.

The present results show that homologous tolerance development to PL is possible in *L. monocytogenes* and that repeatedly treated microorganisms are approximately 1 log CFU cm^-2^ less susceptible to subsequent PL treatments. This is in accordance with Rajkovic et al. (2009). Concerning the stability of this increased tolerance in *L. monocytogenes* in time, discrepancies to Rajkovic et al. (2009) were detected. While the named authors exhibited stability of the induced tolerance at a storage temperature of -75°C, the present study observed a decline of stability at a storage temperature of -30°C. This may be explained by the temperature difference and associated accelerated biochemical processes.

In a study by Massier et al. (2011) the adaptation phenomenon to PL treatment was also demonstrated for strains of *Pseudomonas aeruginosa*. In this context, the authors reported constant mutation frequency but altered abundance of proteins. In detail, *P. aeruginosa* was found capable of down-regulating energy and carbon metabolism as well as redox-homeostasis and cell motility. In contrast, transcription and translation regulators, proteins associated with heat-shock and phage-related proteins were overproduced. The authors interpreted these findings as reallocating resources by limiting energy conversion processes and up-regulating of proteins involved in chaperone mechanisms and probably in response to DNA damages to protect the cell against repeated PL stress.

This combination of findings provides some support for the conceptual premise that homologous, sub-lethal PL stress applied over an extended period of time can induce tolerance development in bacteria. Since these findings, however, obviously are restricted to laboratory conditions, natural occurrence is not documented yet. Nevertheless, the number of studies reporting capability of bacterial populations to build-up tolerance toward novel and mild inactivation treatments has risen the awareness of the scientific community as well as of food business operators in recent years (Yousef and Courtney, 2003; Raso et al., 2005; Rajkovic et al., 2010, 2011; Bradley et al., 2012; Halpin et al., 2014).

To avoid occurrence and persistence of tolerant (sub) populations in the processing environment and, as a consequence, on or in food, Rajkovic et al. (2009, 2011) identified several preventive and control measures. These include appropriate process and equipment design, cleaning and disinfection, sampling program, discourage of the rework and adequate combination of different preservation factors, also known as hurdle technology, during the shelf-life of the product (Leistner and Gorris, 1995; Rajkovic et al., 2011).

### Inactivation Kinetics

In the last decade, there has been an increasing amount of literature on the inactivation kinetic behavior of microorganisms as a result of novel, mild decontamination technologies. In most cases, the log-linear model is described as being not suitable for the description of microbial inactivation (Luksiene et al., 2007; Bialka et al., 2008; Farrell et al., 2010; Keklik et al., 2012; Lasagabaster and de Marañón, 2012; Uesugi et al., 2013).

In order to facilitate a strain specific evaluation of the inactivation kinetics, the present study deliberately used single strains of *L. monocytogenes* instead of a strain mix like applied by Rajkovic et al. (2009). From **Figures 1** and **2** as well as **Table 4** one can presume that the inactivation kinetic of both, *L. monocytogenes* Li-16 and Li-P492, is non-log-linear, exhibiting a substantial decline in the early phase and gradually leveling off, also known as tailing, in the later phase of inactivation treatment, which results in an overall concave upward shape of the curve.

These results are consistent with those of other studies and suggest that the inactivation of bacteria with PL is non-linear with a sigmoid shape (Luksiene et al., 2007; Farrell et al., 2010). The substantial decline and less pronounced shoulder may originate from the fact that the PL treatment of 0.46 J cm^-2^ was already too intense to study the cell injury phase, but adequate to illustrate the rapid decline of surviving cells after a maximum amount of injury and a minimum of additional energy required to cause tremendous cell death rates. Similar inactivation patterns for PL have, for example, been previously shown by Uesugi et al. (2007), Bialka et al. (2008), Keklik et al. (2012) and Lasagabaster and de Marañón (2012).

In order to assess which kinetic model fits best, the Geeraerd and Van Impe inactivation model-fitting tool (GInaFiT) was used, and the two best fitting models, the log-linear + tail as well as the Weibull model, were chosen for comparison with the log-linear model. RMSE values for control, stressed and stored strains of Li-16 and Li-492 are listed in **Table 4**. The log-linear + tail model was apparently more suitable than the linear model for describing the survival curves observed in this study. Similar to the log-linear + tail model, the Weibull model can be also fitted to the curve exhibiting upward concavity. Concavity is proven by the shape parameter *p*(-) which is, in all cases, below the value of 1 (Mafart et al., 2002; Geeraerd et al., 2005; **Table 4**). In most cases, the RMSE values and thus the “average” discrepancy between the (transformed) observed data and their predicted pendants were lowest with the curves observed with the Weibull model, which gives a good indication for the suitability of this model. Exceptions, however, where the log-linear + tail model fitted the data better than the Weibull model were observed (**Table 4**). Taken together, the mean values of the RMSE were 1.186, 0.705, and 0.660 for the log-linear, log-linear + tail and Weibull model, respectively. According to the ANOVA analysis, the mean of RMSE obtained from the log-linear model was significantly (*p* < 0.05) higher than those from the log-linear + tail and Weibull model. A significant difference between the means of RMSE obtained from the log-linear + tail and Weibull model, however, was not detected (*p* > 0.05). Overall, this suggests that of the three tested models the Weibull and the log-linear + tail model are best suited for describing the process of PL inactivation on *L. monocytogenes* Li-16 and Li-P492 inoculated on TSA + YE medium.

The present findings contribute to the general perception that the log-linear inactivation is not suitable for the description of the microbial inactivation pattern (Luksiene et al., 2007; Bialka et al., 2008; Farrell et al., 2010; Keklik et al., 2012; Lasagabaster and de Marañón, 2012; Uesugi et al., 2013). It should be, therefore, considered to use alternative models like the Weibull or log-linear + tail model to describe the inactivation patterns of PL and other novel and mild inactivation technologies. This allows to predict microbial inactivation and to develop optimum and safe processes (Chen and Hoover, 2003; Uesugi et al., 2007; Bialka et al., 2008; Keklik et al., 2012; Lasagabaster and de Marañón, 2012).

### Heat Stress and Cross-Tolerance Development to PL

To date, little is known about the influence of prior food processing conditions (applied sequentially or simultaneously) on the subsequent cross-tolerance of bacteria to non-thermal technologies such as PL (Bradley et al., 2012). In their study, Bradley et al. (2012) investigated the relationship between sub-lethal levels of acid (pH 5.5, 1 h), salt (7.5% NaCl, 1 h) and heat (48°C, 1 h) and the capability of different strains of *L. monocytogenes* to cope with subsequently applied PL stress. The findings suggested that sub-lethal levels of acid and salt significantly reduced the ability to cope with the PL stress. Acid adaptation during growth until stationary phase, however, did not significantly influence the outcome. Likewise, exposure to sub-lethal levels of heat treatment did not significantly affect the PL sensitivity. However, increased invasion of *Caco-2* cells was observed after heat treatment and thus may be linked to modified virulence characteristics of *L. monocytogenes* (Bradley et al., 2012).

The present study showed that *L. monocytogenes* Li-P492 exhibited a significant (*p* < 0.05) higher cross-tolerance behavior following PL treatment of 0.46 J cm^-2^ after previous heat stress (45°C) of approximately 1 log CFU cm^-2^. In detail, the non-heat treated samples exhibited an inactivation of -3.38 ± 0.29, and the 1 and 1.5 h heat treated samples resulted in an inactivation of -2.36 ± 0.03 and -2.44 ± 0.12 log CFU cm^-2^. However, no significant difference between the treatments was detectable (*p* > 0.05).

While these findings stand in contrast to the results reported by Bradley et al. (2012), they may be explained by the time period between heat stress, inoculation and PL treatment (1 h). This could have given the bacteria the opportunity to manifest protection mechanisms as described by Massier et al. (2011). Cross-tolerance development should, therefore, not be neglected in food processing environments.

### Fluence and Temperature Measurement

The temperature measurements revealed that the initial temperature of the samples after inoculation was 18.7 ± 1.1°C and that a significant (*p* = 0.01) increase in temperature was observed with increasing treatment times at the same distance from the quartz window. Further, temperature significantly (*p* = 0.01) increased with a decrease in distance for the same treatment time. At a treatment time of 20 s, the maximum mean temperatures recorded for shelf settings of 3 and 7 were 39.8 ± 0.5 and 32.4 ± 0.7°C, respectively. These findings seem to be consistent with those of other studies and suggest that the PL treatment was conducted under non-thermal conditions. Thereby, the moderate warming of the matrix can be explained by the absorption of the light spectrum emitted by the lamp and the inverse square law (Gómez-López et al., 2005; Keklik et al., 2009, 2010).

The results obtained from the fluence measurements indicated that the total amount of energy obtained by the sample ranged from 0.46 to 20.78 J cm^-2^ (**Table 2**). These results are in agreement with those of Keklik et al. (2009), who reported similar results for a structurally identical PL device.

### TEM Observations of Antimicrobial Actions

**FIGURE 3:**
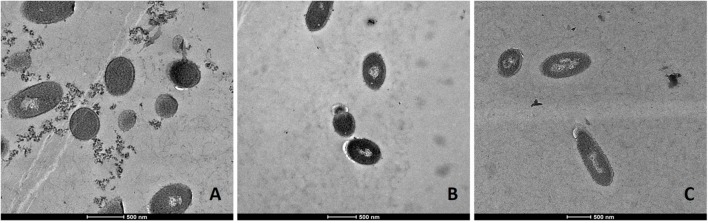
**Transmission electron microscopy (TEM) micrographs of untreated *L. monocytogenes* Li-P69 cells.** Scale bar equals 500 nm **(A–C)**.

**FIGURE 4:**
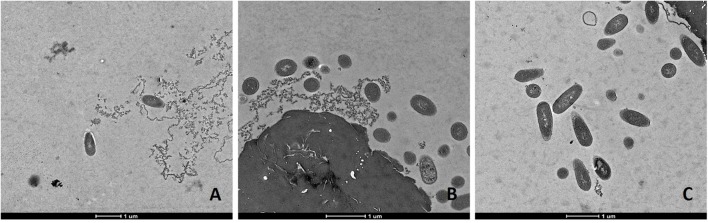
**Transmission electron microscopy (TEM) micrographs of *L. monocytogenes* Li-P69 cells treated with 1.2 J cm^-2^ for 3 s.** Scale bar equals 1 μm **(A–C)**.

**FIGURE 5:**
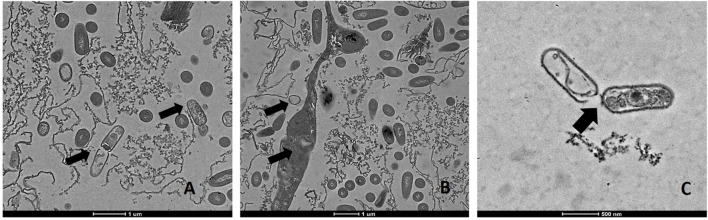
**Transmission electron microscopy (TEM) micrographs of pulsed-light exposed *L. monocytogenes* Li-P69 cells treated with 2.6 J cm^-2^ for 5 s.** Scale bar equals 1 μm **(A,B)** and 500 nm **(C)**. Arrows indicate irreversible damage to bacterial cells, melting structures, and ghost cells (empty envelope structures).

Transmission electron microscopic analysis of bacteria reveal nanometer resolutions to better understand changes of the cellular organizations in relation to food processing technologies. The mechanism of microbial inactivation by PL is mainly based on the UV fraction of the spectrum including the photothermal, -chemical, and physical effect (Ramos-Villarroel et al., 2012). In order to study the morphological effect, cells of Li-P69 were analyzed using TEM. The untreated rod-shaped cells served as control to ensure main differences among treated and untreated samples through imaging. Cells were mainly observed at the population rather than the single-cell level. Control cells showed a homogenous cytoplasm appearance with distinct cell membrane structures (**Figure 3**). Slightly brighter aggregation areas in the middle of the cytoplasm may indicate cell damage, elevated cell age and denaturation through, e.g., sample preparation. After treatment of inoculated cells, a lethal effect could be observed from a cell morphological point of view. Cells are represented by intracytoplasmatic coagulated material (**Figure 4**) maybe resulting from microprecipitation of abnormal proteins and membranes (Díaz-Visurraga et al., 2010). With the increase of the treatment time (3 and 5 s) and the fluence rate, respectively (1.2 and 2.6 J cm^-2^), an elevated antimicrobial effect related to structural differences in the cell compartments (cell wall, cytoplasm) of the Gram-positive *L. monocytogenes* strain could be demonstrated (**Figures 4** and **5**, respectively). The cells were mainly characterized by a cytoplasm shrinkage (**Figures 5A,C**) and release of the cellular content at the peripheral side (**Figure 5C**). Clear extracellular fibrous structures increase by the PL intensity indicating disrupted cell membrane components that are partly folded (**Figures 5A,B**). Further the formation of ghost cells (empty and flaccid cells) characterized by intact cell envelope structure and loss of intracellular material was more frequently observed in the cell population treated for 5 s (**Figure 5**). Filamentation, observed as enlarged cells with indistinguishable cell membrane structure from cytoplasm and melted-like formations were acquired after PL treatment for 5 s (**Figure 5B**). This phenomenon occurs when cell growth continues in the absence of cell division leading to defective organisms and is therefore evidently associated to stressful environments (Díaz-Visurraga et al., 2010). The basically strictly controlled uniformity of cell shape and size may also be altered to the purpose. Filamentation is known for various foodborne pathogens but the biological role is not fully understood so far (Justice et al., 2008). In a study of Vail et al. (2012) the proportion and length of filaments of *L. monocytogenes* increased up to 8.5 times after application of food associated stress conditions (e.g., NaCl, pH) while the CFU values decreased. This could have relevant implications for food safety as filaments form single colonies but may divide into individual cells after stress removal.

Alterations in cells caused by PL have been shown in other studies with regard to Gram-negative and –positive bacteria, bacterial endospores and yeast cells (Ramos-Villarroel et al., 2012). The so called multitarget properties of PL caused by photothermal and photophysical effects are partly proven for *L. monocytogenes* and *E. coli* O157:H7 under TEM observations (Cheigh et al., 2012).

However, the introduction of artifacts should be integrated into image interpretation and be handled with care (Díaz-Visurraga et al., 2010).

## Conclusion

This study provides some evidence for homologous tolerance development in *L. monocytogenes* against repeated PL stress as well as heterologous tolerance development from heat stress to PL. Further, the kinetic analysis confirmed that the log-linear model is not suited to describe the PL inactivation of *L. monocytogenes* and that the Weibull or the log-linear + tail model should be used. The models could be, therefore, used to assess microbial reduction or to optimize the treatment for a target reduction. TEM images showed remarkable cytoplasmic damage in cells and disintegrations of the morphological structure with increased fluence rate.

Both the tolerance development and the inactivation kinetics should be taken into account when substituting conventional inactivation treatments like heat by novel and mild inactivation technologies or combined treatments following a hurdle concept. Hence, future research should focus on the effect of tolerance development in pathogenic microorganisms and its relevance for food safety. This amongst others should include demonstration and quantification of this phenomenon *in vitro* as well as under real processing conditions.

## Author Contributions

MZ: Project leader of pulsed-light project; experimental design and setup; article writing and final approval of the version to be published; agreement to be accountable for all aspects of the work in ensuring that questions related to the accuracy or integrity of any part of the work are appropriately investigated and resolved; TEM examinations and interpretations. VH: Doctorate student in this project; practical work in the lab and design of experiments; preparation of protocols; substantial contribution to the conception or design of the work; article writing of the main content, therefore first author. WK: Co-Project-leader of Pulsed-light project; mentoring; final approval of the version to be published; agreement to be accountable for all aspects of the work in ensuring that questions related to the accuracy or integrity of any part of the work are appropriately investigated and resolved. HM: Student practical work in the laboratory; sample preparation for TEM analysis and protocol development; kinetic analysis. AP: Pre-experiment phase; student practical work in the laboratory; sample preparation for TEM analysis and protocol development; kinetic analysis. AL: Doctorate student; TEM experiments setup. ER: supervisor TEM experiments.

## Conflict of Interest Statement

The authors declare that the research was conducted in the absence of any commercial or financial relationships that could be construed as a potential conflict of interest.

## References

[B1] Archer D. (1996). Preservation microbiology and safety: evidence that stress enhances virulence and triggers adaptive mutations. *Trends Food Sci. Technol.* 7 91–95. 10.1016/0924-2244(96)81303-3

[B2] Aymerich T. Picouet P. A. Monfort J. M. (2008). Decontamination technologies for meat products. *Meat Sci.* 78 114–129. 10.1016/j.meatsci.2007.07.007 22062101

[B3] Bialka K. L. Walker P. N. Puri V. M. Demirci A. (2008). Pulsed UV-light penetration of characterization and the inactivation of *Escherichia coli* K12 in solid model systems. *Trans. ASABE* 51 195–204. 10.13031/2013.24204

[B4] Bigelow W. D. Esty J. R. (1920). The thermal death point in relation to typical thermophilic organisms. *J. Infect. Dis.* 27 602–617. 10.1093/infdis/27.6.602

[B5] Bradley D. McNeil B. Laffey J. G. Rowan N. J. (2012). Studies on the pathogenesis and survival of different culture forms of *Listeria monocytogenes* to pulsed UV-light irradiation after exposure to mild-food processing stresses. *Food Microbiol.* 30 330–339. 10.1016/j.fm.2011.12.024 22365345

[B6] Butz P. Tauscher B. (2002). Emerging technologies: chemical aspects. *Food Res. Int.* 35 279–284. 10.1016/S0963-9969(01)00197-1

[B7] Cerf O. (1977). A review. Tailing of survival curves of bacterial spores. *J. Appl. Microbiol.* 42 1–19. 10.1111/j.1365-2672.1977.tb00665.x 323208

[B8] Cheigh C. I. Park M. H. Chung M. S. Shin J. K. Park Y. S. (2012). Comparison of intense pulsed light- and ultraviolet (UVC)-induced cell damage in *Listeria monocytogenes* and *Escherichia coli* O157:H7. *Food Control.* 25 654–659. 10.1016/j.foodcont.2011.11.032

[B9] Chen H. Hoover D. G. (2003). Modeling the combined effect of high hydrostatic pressure and mild heat on the inactivation kinetics of *Listeria monocytogenes* Scott A in whole milk. *Innov. Food Sci. Emerg.* 4 25–34. 10.1016/S1466-8564(02)00083-8

[B10] Cleaver J. F. (2003). Photoreactivation. *DNA Repair* 2 629–638. 10.1016/S1568-7864(03)00037-5 12713819

[B11] Cole M. B. Davies K. W. Munro G. Holyoak C. D. Kilsby D. C. (1993). A vitalistic model to describe the thermal inactivation of *Listeria monocytogenes*. *J. Ind. Microbiol.* 12 232–239. 10.1007/BF01584195

[B12] Díaz-Visurraga J. Cárdenas G. García A. (2010). “Morphological changes induced in bacteria as evaluated by electron microscopy,” in *Microscopy: Science, Technology, Applications and Education*. eds Méndez-Vilas A. Díaz J. (Badajoz: Formatex), 307–315.

[B13] Dunn J. E. Clark R. W. Asmus J. F. Pearlman J. S. Boyer K. Painchaud F. (1989). *Methods for Preservation of Foodstuffs.* San Diego, CA: Maxwell Laboratories Inc. US Patent 4871559.

[B14] European Food Safety Authority, and European Centre for Disease Prevention, and Control [EFSA and ECDC] (2015). The european union summary report on trends, and sources of zoonoses, zoonotic agents, and food-borne outbreaks. *EFSA J.* 13:3991. 10.2903/j.efsa.2018.5500 PMC7009540 32625785

[B15] European Union Reference Laboratory for Listeria monocytogenes [EURL Lm] (2014). *EURL Lm Technical Guidance Document for Conducting Shelf-life Studies on Listeria monocytogenes in Ready-to-Eat Foods. Version 3–6.* Available at: http://www.evira.fi/files/attachments/fi/laboratoriotoiminta/vertailulaboratoriot/eurl_lm_technical_guidance_document_lm_shelf-life_studies_v3_2014-06-06.pdf (accessed June, 2014).

[B16] Farrell H. P. Garvey M. Cormican M. Laffey J. G. Rowan N. J. (2010). Investigation of critical inter-related factors affecting the efficacy of pulsed light for inactivating clinically relevant bacterial pathogens. *J. Appl. Microbiol.* 108 1494–1508. 10.1111/j.1365-2672.2009.04545.x 19796119

[B17] Food and Drug Administration [FDA] (1996). *Code of Federal Regulations (CFR) Title 21 Part 179. Irradiation in the Production, Processing and Handling of Food.* Washington, DC: Food and Drug Administration.

[B18] Francois K. Devlieghere F. Uyttendaele M. Debebere J. (2006). Risk assessment of *Listeria monocytogenes*: impact of individual cell variability on the exposure assessment step. *Risk Anal.* 26 105–114. 10.1111/j.1539-6924.2006.00716.x 16492184

[B19] Geeraerd A. H. Herremans C. H. Van Impe J. F. (2000). Structural model requirementsw to describe microbial inactivation during a mild heat treatment. *Int. J. Food Microbiol.* 59 185–209. 10.1016/S0168-1605(00)00362-7 11020040

[B20] Geeraerd A. H. Valdramidis V. P. Van Impe J. F. (2005). GInaFiT, a freeware tool to assess non-log-linear microbial survivor curves. *Int. J. Food Microbiol.* 102 95–105. 10.1016/j.ijfoodmicro.2004.11.038 15893399

[B21] Gómez-López V. M. Devlieghere F. Bonduelle V. Debevere J. (2005). Factors affecting the inactivation of micro-organisms by intense light pulses. *J. Appl. Microbiol.* 99 460–470. 10.1111/j.1365-2672.2005.02641.x 16108787

[B22] Halpin R. M. Duffy L. Cregenzán-Alberti O. Lyng J. G. Noci F. (2014). The effect of non-thermal processing technologies on microbial inactivation: an investigation into sub-lethal injury of *Escherichia coli* and *Pseudomonas* fluorescens. *Food Control* 41 106–115. 10.1016/j.foodcont.2014.01.011

[B23] Hauben K. J. A. Bartlett D. H. Soontjens C. C. F. Cornelis K. Wuytack E. Y. Michielis C. W. (1997). *Escherichia coli* mutants resistant to inactivation by high hydrostatic pressure. *Appl. Environ. Microbiol.* 63 945–950. 9055412 10.1128/aem.63.3.945-950.1997 PMC168386

[B24] Haughton P. N. Lyng J. G. Morgan D. J. Cronin D. A. Fanning S. Whyte P. (2011). Efficacy of high-intensity pulsed light for the microbiological decontamination of chicken, associated packaging, and contact surfaces. *Foodborne Pathog. Dis.* 8 109–117. 10.1089/fpd.2010.0640 20932088

[B25] Havelaar A. H. Brul S. de Jong A. de Jonge R. Zwietering M. H. ter Kuile B. H. (2010). Future challenges to microbial food safety. *Int. J. Food Microbiol.* 139 S79–S94. 10.1016/j.ijfoodmicro.2009.10.015 19913933

[B26] Heinrich V. Zunabovic M. Varzakas T. Bergmair J. Kneifel W. (2016). Pulsed light treatment of different food types with a special focus on meat: a critical review. *Crit. Rev. Food Sci. Nutr.* 56 591–613. 10.1080/10408398.2013.826174 25575192

[B27] Hill C. Cotter P. D. Sleator R. D. Gahan C. G. M. (2002). Bacterial stress response in *Listeria monocytogenes*: jumping the hurdles imposed by minimal processing. *Int. Dairy J.* 12 273–283. 10.1016/S0958-6946(01)00125-X

[B28] Huang L. (2009). Thermal inactivation of *Listeria monocytogenes* in ground beef under isothermal and dynamic temperature conditions. *J. Food Eng.* 90 380–387. 10.1016/j.jfoodeng.2008.07.011

[B29] Justice S. S. Hunstad D. A. Cegelski L. Hultgren S. J. (2008). Morphological plasticity as a bacterial survival strategy. *Nat. Rev. Microbiol.* 6 162–168. 10.1038/nrmicro1820 18157153

[B30] Keklik N. M. Demirci A. Puri V. M. (2009). Inactivation of *Listeria monocytogenes* on unpackaged and vacuum-packaged chicken frankfurters using pulsed UV-light. *J. Food Sci.* 74 M431–M439. 10.1111/j.1750-3841.2009.01319.x 19799670

[B31] Keklik N. M. Demirci A. Puri V. M. (2010). Decontamination of unpackaged and vacuum-packaged boneless chicken breast with pulsed ultraviolet light. *Poult. Sci.* 89 570–581. 10.3382/ps.2008-00476 20181876

[B32] Keklik N. M. Demirci A. Puri V. M. Heinemann P. H. (2012). Modeling the inactivation of *Salmonella* Typhimurium, *Listeria monocytogenes*, and *Salmonella* Enteritidis on poultry products exposed to pulsed UV light. *J. Food Prot.* 75 281–288. 10.4315/0362-028X.JFP-11-298 22289588

[B33] Knorr D. Froehling A. Jaeger H. Reineke K. Schlueter O. Schoessler K. (2011). Emerging technologies in food processing. *Annu. Rev. Food Sci. Technol.* 2 203–235. 10.1146/annurev.food.102308.124129 22129381

[B34] Krishnamurthy K. Tewari J. C. Irudayaraj J. Demirci A. (2010). Microscopic and spectroscopic evaluation of inactivation of *Staphylococcus aureus* by pulsed UV light and infrared heating. *Food Bioprocess Technol.* 3 93–104. 10.1007/s11947-008-0084-8

[B35] Lasagabaster A. de Marañón I. M. (2012). Sensitivity to pulsed light technology of several spoilage and pathogenic bacteria isolated from fish products. *J. Food Prot.* 75 2039–2044. 10.4315/0362-028X.JFP-12-071 23127714

[B36] Leistner L. Gorris L. G. M. (1995). Food preservation by hurdle technology. *Trends Food Sci. Technol.* 6 41–46. 10.1016/S0924-2244(00)88941-4

[B37] Lin Y.-D. Chou C.-C. (2004). Effect of heat shock on thermal tolerance and susceptibility of *Listeria monocytogenes* to other environmental stresses. *Food Microbiol.* 21 605–610. 10.1016/j.fm.2003.10.007

[B38] Lou Y. Q. Yousef A. E. (1997). Adaption to sublethal environmental stresses protects *Listeria monocytogenes* against lethal preservation factors. *Appl. Environ. Microbiol.* 63 1252–1255. 9097420 10.1128/aem.63.4.1252-1255.1997 PMC168417

[B39] Luksiene Z. Gudelis V. Buchovec I. Raudeliuniene J. (2007). Advanced high-power pulsed light device to decontaminate food from pathogens: effects on *Salmonella* typhimurium viability in vitro. *J. Appl. Microbiol.* 103 1545–1552. 10.1111/j.1365-2672.2007.03403.x 17953565

[B40] Mafart P. Couvert O. Gaillard S. Leguerinel I. (2002). On calculating sterility in thermal preservation methods: application of the Weibull frequency distribution model. *Int. J. Food Microbiol.* 72 107–113. 10.1016/S0168-1605(01)00624-9 11843401

[B41] Marquenie D. Geeraerd A. H. Lammertyn J. Soontjens C. Van Impe J. F. Michielis C. W. (2003). Combinations of pulsed white light and UV-C or mild heat treatment to inactivate conidia of Botrytis cinerea and Monilia fructigena. *Int. J. Food Microbiol.* 85 185–196. 10.1016/S0168-1605(02)00538-X 12810282

[B42] Massier S. Rincé A. Maillot O. Feuilloley M. G. J. Orange N. Chevalier S. (2011). Adaption of *Pseudomonas aeruginosa* to a pulsed light-induced stress. *J. Appl. Microbiol.* 112 502–511. 10.1111/j.1365-2672.2011.05224.x 22188372

[B43] Ortega-Rivas E. (2012). *Non-Thermal Food Engineering Operations*, Chap. 12. Berlin: Springer Science+Business Media.

[B44] Palmieri L. Cacace D. (2005). “High intensity pulsed light technology,” in *Emerging Technologies for Food Processing*, ed. Sun D.-W. (London: Elsevier Academic Press), 279–306.

[B45] Peleg M. Cole M. B. (1998). Reinterpretation of microbial survival curves. *Crit. Rev. Food Sci. Nutr.* 38 353–380. 10.1080/10408699891274246 9704188

[B46] Rajkovic A. Smigic N. Devlieghere F. (2010). Contemporary strategies in combating microbial contamination in food chain. *Int. J. Food Microbiol.* 141 S29–S42. 10.1016/j.ijfoodmicro.2009.12.019 20056287

[B47] Rajkovic A. Smigic N. Devlieghere F. (2011). Growth of *Escherichia coli* O157:H7 and *Listeria monocytogenes* with prior resistance to intense pulsed light and lactic acid. *Food Microbiol.* 28 869–872. 10.1016/j.fm.2010.12.002 21569928

[B48] Rajkovic A. Smigic N. Uyttendaele M. Medic H. De Zutter L. Devlieghere F. (2009). Resistance of *Listeria monocytogenes*, *Escherichia coli* O157:H7 and *Campylobacter jejuni* after exposure to repetitive cycles of mild bactericidal treatments. *Food Microbiol.* 26 889–895. 10.1016/j.fm.2009.06.006 19835777

[B49] Ramos-Villarroel A. Y. Aron-Maftei N. Martín-Belloso O. Soliva-Fortuny R. (2012). The role of pulsed light spectral distribution in the inactivation of *Escherichia coli* and *Listeria innocua* on fresh-cut mushrooms. *Food Control.* 24 206–213. 10.1016/j.foodcont.2011.09.029

[B50] Raso J. Pagan R. Condon S. (2005). “Nonthermal technologies in combination with other preservation factors,” in *Novel Food Processing Technologies*, eds Barbosa-Cánovas G. V. Tapia M. S. Cano M. P. Belloso O. M. Martinez A. (New York, NY: Marcel Dekker).

[B51] Ratkowsky D. A. (2003). “Model fitting and uncertainty,” in *Modeling Microbial Responses in Foods*, eds McKellar R. Lu X. (Boca Raton, FL: CRC Press), 151–196.

[B52] Ringus D. L. Moraru C. I. (2013). Pulsed light inactivation of *Listeria innocua* on food packaging materials of different surface roughness and reflectivity. *J. Food Eng.* 114 331–337. 10.1016/j.jfoodeng.2012.08.022

[B53] Rowan N. J. (1999). Evidence that inimical food-preservation barriers alter microbial resistance, cell morphology and virulence. *Trends Food Sci. Technol.* 10 261–270. 10.1016/S0924-2244(99)00060-6

[B54] Rowan N. J. MacGregor S. J. Anderson J. G. Fouracre R. A. McIllvaney L. Farish O. (1999). Pulsed-light inactivation of food-related microorganisms. *Appl. Environ. Microbiol.* 65 1312–1315. 10049899 10.1128/aem.65.3.1312-1315.1999 PMC91180

[B55] Rowan N. J. Valdramidis V. P. Gómez-López V. M. (2015). A review of quantitative methods to describe efficacy of pulsed light generated inactivation data that embraces the occurrence of viable but not culturable state microorganisms. *Trends Food Sci. Technol.* 44 79–92. 10.1016/j.tifs.2015.03.006

[B56] Sofos J. N. (2005). *Improving the Safety of Fresh Meat.* Cambridge: Woodhead Publishing Limited.

[B57] Sofos J. N. (2008). Challenges to meat safety in the 21st century. *Meat Sci.* 78 3–13. 10.1016/j.meatsci.2007.07.027 22062090

[B58] The Rapid Alerst System for Food, and Feed [RASFF] (2014). *Annual Report.* Luxembourg: Publication of the European Union.

[B59] Uesugi A. R. Hsu L. Moraru C. I. (2013). Effect of pulsed light treatments on the growth and resistance behavior of *Listeria monocytogenes* 10403S, *Listeria innocua*, and *Escherichia coli* ATCC 25922 in a liquid substrate. *J. Food Prot.* 76 435–439. 10.4315/0362-028X.JFP-12-370 23462080

[B60] Uesugi A. R. Woodling S. E. Moraru C. I. (2007). Inactivation kinetics and factors of variability in the pulsed light treatment of *Listeria innocua* cells. *J. Food Prot.* 70 2518–2525. 18044429 10.4315/0362-028x-70.11.2518

[B61] Vail K. M. McMullen L. M. Jones T. H. (2012). Growth and filamentation of cold-adapted, log-phase *Listeria monocytogenes* exposed to salt, acid, or alkali stress at 3 degrees C. *J. Food Prot.* 75 2142–2150. 10.4315/0362-028X.JFP-12-199 23212010

[B62] Wang T. Macgregor S. J. Anderson J. G. Woolsey G. A. (2005). Pulsed ultra-violet inactivation spectrum of *Escherichia coli*. *Water Res.* 39 2921–2925. 10.1016/j.watres.2005.04.067 15993922

[B63] Weiss J. Gibis M. Schuh V. Salminen H. (2010). Advances in ingredient and processing systems for meat and meat products. *Meat Sci.* 86 196–213. 10.1016/j.meatsci.2010.05.008 20619800

[B64] Yousef A. E. Courtney P. D. (2003). “Basics of stress adaption and implications in new-generation foods,” in *Microbial Stress Adaption and Food Safety*, eds Yousef A. E. Juneja V. K. (Boca Raton, FL: CRC Press LLC).

